# Clinical Diagnosis and Early Medical Management for Endometriosis: Consensus from Asian Expert Group

**DOI:** 10.3390/healthcare10122515

**Published:** 2022-12-12

**Authors:** Mee-Ran Kim, Charles Chapron, Thomas Römer, Angela Aguilar, Amphan Chalermchockcharoenkit, Siddharta Chatterjee, Le Thi Anh Dao, Yoke Fai Fong, Hendy Hendarto, Syarief Taufik Hidayat, Su Yen Khong, Li Ma, Pratap Kumar, Relly Yanuari Primariawan, Anthony Siow, Areepan Sophonsritsuk, Ramani Devi Thirunavukarasu, Bui Chi Thuong, Chih-Feng Yen

**Affiliations:** 1 Department of Obstetrics & Gynecology, College of Medicine, The Catholic University of Korea, Seoul 06591, Republic of Korea; 2 Université Paris Descartes, Sorbonne Paris Cité, Faculté de Médecine, Assistance Publique–Hôpitaux de Paris (AP-HP), Hôpital Universitaire Paris Centre (HUPC), Centre Hospitalier Universitaire (CHU) Cochin, Department of Gynecology Obstetrics II and Reproductive Medicine, 75014 Paris, France; 3 Evangelisches Klinikum Köln Weyertal, Department of Obstetrics & Gynecology, Academic Hospital of the University, 50931 Cologne, Germany; 4 University of the Philippines College of Medicine, The Philippine General Hospital, Manila 1000, Philippines; 5 Department of Obstetrics & Gynaecology, Faculty of Medicine Siriraj Hospital, Mahidol University, Bangkok 10700, Thailand; 6 Calcutta Fertility Mission, Kolkata 700019, India; 7 Department of Obstetrics & Gynecology, Hanoi Medical University, Hanoi 116001, Vietnam; 8 National University of Singapore, Singapore 119260, Singapore; 9 Department of Obstetrics & Gynecology, Faculty of Medicine, Universitas Airlangga/Dr. Sutomo Academic Hospital, Surabaya 60111, Indonesia; 10 Department of Obstetrics & Gynecology of Kariadi General Hospital Medical Centre, Faculty of Medicine Diponegoro University, Semarang 50244, Indonesia; 11 Subang Jaya Medical Centre, Kuala Lumpur 47500, Malaysia; 12 National University Hospital, Singapore 119074, Singapore; 13 Kasturba Medical College, Manipal Academy of Higher Education, Manipal 576104, India; 14 Obstetrics & Gynecology Department, Dr. Soetomo General Hospital-School of Medicine of Airlangga University, Surabaya 60286, Indonesia; 15 ASC Clinic for Women Pte Ltd., Gleneagles Hospital, Singapore 258500, Singapore; 16 Faculty of Medicine, Ramathibodi Hospital, Mahidol University, Bangkok 10400, Thailand; 17 Ramakrishna Medical Centre LLP, Trichy 620003, India; 18 University of Medicine and Pharmacy at Ho Chi Minh City, Ho Chi Minh City 700000, Vietnam; 19 Department of Obstetrics & Gynecology, Chang Gung Memorial Hospital (Linkou), Chang Gung University College of Medicine, Taoyuan 333, Taiwan

**Keywords:** endometriosis, diagnosis, medical management

## Abstract

This work provides consensus guidance regarding clinical diagnosis and early medical management of endometriosis within Asia. Clinicians with expertise in endometriosis critically evaluated available evidence on clinical diagnosis and early medical management and their applicability to current clinical practices. Clinical diagnosis should focus on symptom recognition, which can be presumed to be endometriosis without laparoscopic confirmation. Transvaginal sonography can be appropriate for diagnosing pelvic endometriosis in select patients. For early empiric treatment, management of women with clinical presentation suggestive of endometriosis should be individualized and consider presentation and therapeutic need. Medical treatment is recommended to reduce endometriosis-associated pelvic pain for patients with no immediate pregnancy desires. Hormonal treatment can be considered for pelvic pain with a clinical endometriosis diagnosis; progestins are a first-line management option for early medical treatment, with oral progestin-based therapies generally a better option compared with combined oral contraceptives because of their safety profile. Dienogest can be used long-term if needed and a larger evidence base supports dienogest use compared with gonadotropin-releasing hormone agonists (GnRHa) as first-line medical therapy. GnRHa may be considered for first-line therapy in some specific situations or as short-term therapy before dienogest and non-steroidal anti-inflammatory drugs as add-on therapy for endometriosis-associated pelvic pain.

## 1. Introduction

Endometriosis is an estrogen-dependent, progesterone-resistant gynecologic condition characterized by the presence of ectopic endometrial-like tissue outside the uterine cavity; endometriosis is strongly affected by cyclic changes in response to steroid hormones and is associated with an inflammatory response in the peritoneal cavity [1,2,3]. Endometriosis is characterized by chronic pelvic pain, with common clinical presentations of dysmenorrhea, dyspareunia, dyschezia, dysuria, and infertility [1,4]. Accordingly, it is an important cause of morbidity that can detrimentally affect the quality of life (QoL) in women of reproductive age [5,6]. 

The exact etiology and pathogenesis of endometriosis continue to be elucidated, with environmental and genetic factors implicated [4,7,8,9]. Similarly, the true burden of endometriosis is unknown although the prevalence rate in Western populations is estimated to range from 2% to 10% of women of reproductive age, with an estimated 50% of infertile women affected [10,11]. The burden of endometriosis in the Asian population has been poorly characterized but is considered in some studies to be greater in Asian women than women from other continents [12,13]. 

Delay in diagnosis of endometriosis is commonly reported, some as long as 11 years [10,14,15,16,17,18,19,20]. Studies of diagnostic delays in Asia are less common, but it is possible that diagnosis in Asia may occur earlier including because of cultural and socioeconomic barriers limiting access to care. These delays can result in ongoing symptoms that detrimentally affect QoL and fertility [14]. Limitations in current approaches for diagnosis of endometriosis may be contributing to these delays. 

The diagnosis and treatment of endometriosis has undergone considerable changes in recent years with an increasing focus on patient-centered care that includes more frequent clinical diagnosis and early medical management [14,21]. Additionally, improved understanding of the underlying associated hormonal and inflammatory abnormalities and therapeutic targets for endometriosis has led to the availability of new treatments [22,23]. While this changing paradigm for clinical diagnosis and medical management of endometriosis necessitates consideration of how best to deliver patient-centered care, available guidelines and recommendations do not necessarily reflect current practice and the emerging evidence base, including within Asia. 

In this context, a group of clinicians from Asia and Europe with expertise in the diagnosis and treatment of endometriosis met to critically evaluate recent international guidelines and consensus reports [as summarized in Table 1 [7,8,10,22,24,25,26,27]. Literature on clinical diagnosis and early medical management of endometriosis and their applicability to current clinical practice, with a predominant focus within Asia, was also considered. This work is a summation of these deliberations and provides consensus guidance regarding clinical diagnosis and early medical management of endometriosis within Asia. 

## 2. Clinical Diagnosis

### 2.1. Consensus: Focus Should Be Directed towards the Recognition of Symptoms That May Lead to the Diagnosis of Endometriosis, Such as Abdominal-Pelvic Pain and Infertility. These Symptoms Can Be Presumed to Be Endometriosis without the Need for Laparoscopy

Definitive diagnosis of endometriosis relied previously on laparoscopic findings with histological verification [7,10,22,25,26]. However, limitations of laparoscopy include surgical risks and reliance on identifiable pelvic lesions rather than consideration of endometriosis as a systemic disease with variable presentations [14]. While endometriosis is commonly defined histologically, the presence of lesions does not preclude other causes for patients’ symptoms. Conversely, the lack of clinically identified lesions does not necessarily exclude a diagnosis of endometriosis [28]. Further, endometriosis cannot be identified currently by pathogenic features or biomarkers, and the key symptoms of endometriosis like pain and infertility can mistakenly be attributed to other causes.

We propose instead focusing on the patient and her clinical symptoms, which can decrease diagnostic delay in women from low resource settings. A clinical approach to diagnosis also considers that endometriosis can occur without pelvic pain symptoms and that pelvic pain might be attributed to causes other than endometriosis [21,29]. 

The presence of symptoms suggestive of endometriosis warrants further investigation to support diagnosis. Importantly, diagnosis of endometriosis should not be predominantly focused on pain, as the perception of pain is subjective and varies globally [30], but rather clinicians should exert efforts to recognize both gynecologic and non-gynecologic symptoms of endometriosis. Endometriosis should be suspected in reproductive-aged women with chronic and/or cyclic pelvic pain (e.g., dysmenorrhea, deep dyspareunia, dyschezia), pelvic mass (e.g., ovarian endometrioma and adenomyosis), and/or subfertility. It should also be suspected for unexplained fatigue, weariness, depression, anxiety, hematuria, rectal bleeding, and other catamenial symptoms outside the genitourinary system. Patient history is another important consideration in the diagnosis of endometriosis, with infertility history in conjunction with clinical signs and symptoms being strongly associated with endometriosis [11,31,32,33,34,35]. Other patient characteristics suggestive of endometriosis include previous laparoscopic diagnosis or a positive family history [12,16,33,36]. 

Women with endometriosis are diagnosed typically in their 20s and 30s [19,37], but endometriosis should also be considered in adolescents suffering from intractable pain unresponsive to non-steroidal anti-inflammatory drugs (NSAIDs) [38]. This is especially so in adolescents with a strong family history [38]. Thus, adolescents should be encouraged to report their experience with menstruation, as normalizing painful menstruation among this age group may result in delayed diagnosis. 

Physical examination findings are able to identify endometriosis with high accuracy dependent on location of the lesions [39,40,41] and should be included as a component of clinical diagnosis. Inspection and palpation of the abdomen, and depending on patient age and sexual history, physical examination of the pelvis is recommended to identify abdominal masses and pelvic symptoms (e.g., decreased organ mobility and enlargement, visible vaginal lesions, nodules in the posterior vaginal fornix, retroverted uterus) [42]. Pelvic examination should include speculum examination and vaginal palpation, investigation of the position, mobility, size, fixation, or tenderness of the uterus, and evaluation of pelvic tenderness [14,43]. Rectovaginal examination or palpation of the contents of the pouch of Douglas (POD), which can sensitively detect deep endometriosis (DE), should be considered [28]. Notably, the sensitivity and specificity of the pelvic examination depend on only palpable lesions (e.g., ovarian endometriomas enlarged beyond normal ovarian volume and cul de sac or POD masses noted on rectovaginal examination) and may be insufficiently sensitive for other phenotypes [39,40,41].

### 2.2. Consensus: Transvaginal Sonography Is an Appropriate Imaging Technique in the Diagnosis of Pelvic Endometriosis

Imaging must now be considered as a major component of clinical diagnosis to further investigate underlying symptoms, localize the disease, and determine disease severity of endometriosis [21]. Imaging can be used to detect endometrioma, ovarian cysts, and other nodules, masses, and pelvic disorders [7,14,44]. However, its accuracy for the assessment of some pathologies, such as superficial lesions and ovarian foci, is limited [14,44,45].

For some endometriosis subtypes, transvaginal sonography (TVS) improves accuracy when used in conjunction with symptoms, patient history, and/or physical findings [14,39,46]. The International Deep Endometriosis Analysis (IDEA) group has provided 4 basic steps to be used for the sonographic examination of suspected endometriosis including evaluation or assessment of (i) the uterus and adnexa to identify signs of adenomyosis or the presence of endometrioma; (ii) TVS markers such as site-specific tenderness and ovarian mobility; (iii) the status of the POD; and (iv) the presence of DE nodules in the anterior and posterior compartments [47]. Another important consideration with TVS is that it has to be performed by highly experienced sonologists, as ultrasound findings are highly operator dependent [10]. However, proficiency for this technique can be achieved after examining less than 50 patients [48].

Consideration of the appropriateness of TVS for individual patients is required. For those in whom TVS is not appropriate, use of alternative imaging approaches, such as transabdominal or transrectal sonography (TRS), should be considered. TRS may be a sensitive and useful approach, particularly in Asia where cultural norms may make this imaging technique more appropriate. In a diagnostic accuracy study conducted in Asian patients with symptoms of endometriosis, the sensitivity of TRS in diagnosing DE was comparable to that of TVS and magnetic resonance imaging, although its cost limits its utilization in low resource settings [49].

## 3. Early Empiric Medical Management

### 3.1. Consensus: Management of Women with a Presumptive Clinical Presentation Suggestive of Endometriosis Depends on the Individual Patient and Should Consider Her Presentation at That Time and the Need for Therapy

A definitive diagnosis is not required before commencing treatment in patients with pelvic pain who are not desirous of immediate pregnancy. A large percentage of adolescents with chronic pelvic pain or dysmenorrhea are reported to have endometriosis on laparoscopy [50]. The detrimental effect of endometriosis on patient well-being, ovarian reserve, and QoL [5,6,21] further emphasizes the importance of initiating treatment even in the absence of histologic diagnosis of endometriosis. 

We recommend that the primary focus of endometriosis treatment should be the management of a patient’s presenting symptoms. Furthermore, treatment should also be individually tailored, accounting for patient- and disease-related factors (e.g., age, disease severity and extent, fertility requirements, contraception, patient wishes) and treatment-related characteristics (e.g., side effects, compliance, cost) [51]. 

### 3.2. Consensus: Medical Treatment Is Recommended to Reduce Endometriosis-Associated Pelvic Pain for Patients Who Have No Immediate Desire for Pregnancy

Medical therapies for endometriosis induce atrophy within hormonally dependent ectopic endometrium, leading to a decrease in the number and size of lesions [52], ultimately controlling pain and suppressing the hormonally active endometriotic tissue [23]. As endometriosis is an estrogen-dependent systemic disease [1,2,3], we recommend that women with symptoms presumed to be due to endometriosis receive medical therapy, which includes hormonal (e.g., combined oral contraceptives [COCs], gonadotropin-releasing hormone [GnRH] agonists/antagonists, and progestins or anti-progestins) medications. Non-hormonal (e.g., NSAIDs) medications can also be considered, with the choice of therapy dependent on tolerability profile, cost, availability, and patient characteristics.

### 3.3. Consensus: Hormonal Treatment Is a More Effective Option in the Treatment of Pelvic Pain from Clinically Diagnosed Endometriosis. Progestins Are among the First-Line Management Options for Early Medical Treatment

Hormonal treatment for women with suspected or confirmed endometriosis can have a beneficial effect on pain and is not associated with a detrimental effect on subsequent fertility [24]. Of the available hormonal treatments, we consider progestins one of the first-line treatment options for early medical management of endometriosis.

The effect of progesterone on the pathophysiology of endometriosis is multifactorial [23], leading to the development of several progestin-based therapies for the medical treatment of endometriosis. Progestins are believed to exert their effects by decidualization followed by atrophy of endometrial tissue, with suppression of matrix metalloproteinases and angiogenesis proposed as contributory mechanisms [22,23]. The effect of progesterone on inflammatory pathways has also been reported [53]. For a subset of women, impaired action of progesterone on the endometrium may render some hormonal treatments ineffective, although synthetic progestins may overcome this resistance through effects on progesterone receptors and proinflammatory cytokines [54].

Based on a 2012 systematic review of 13 randomized controlled trials (RCTs), progestins or anti-progestins (i.e., medroxyprogesterone acetate, dienogest, cyproterone acetate, norethisterone acetate, danazol) were found to reduce endometriosis-associated pain compared with other interventions, placebo, or no treatment [52]. Notably, most RCTs assessing the treatment of endometriosis-associated pain were conducted in patients with laparoscopically diagnosed endometriosis and side effect profiles of treatments have confounded results [22,26]. Large placebo effects have also been observed and evaluation of treatment durations of 6 months or longer are limited, although such long-term data are emerging as described below. Accordingly and based on current evidence, several guidelines recommend that effective treatment of endometriosis-associated pain can be achieved with progestins in women with suspected or confirmed endometriosis and without a detrimental effect on subsequent fertility [7,10,24,26]. 

The tolerability profile of progestins is another important attribute of the medication, as better tolerated progestins may be more appropriate for long-term use because of the chronicity of the condition. Therefore, we recommend that the differing tolerability profiles of progestins and anti-progestins be considered when selecting a particular medication, including transient (e.g., vaginal bleeding, weight gain, headache, mood change, decreased libido) and irreversible (e.g., thrombosis) adverse effects [10,26]. Additionally, we recommend that women of reproductive age requiring treatment be encouraged to start treatment with progestins to preserve fertility potential as available non-hormonal medical therapies do not suppress the progression of endometriosis [8,10,22,55].

### 3.4. Consensus: Oral Progestin-Based Therapies Are Generally a Better Option Compared with COCs Because of Their Safety Profile

Hormonal contraceptives exert their effects in endometriosis through ovarian and pituitary suppression, or through a general suppression of the hypothalamic–pituitary–ovarian (HPO) axis; estrogen and progesterone combinations or progestins alone lead to decidualization of the endometriotic tissue and decreased disease activity [22,23]. COCs decrease endometriosis-associated dyspareunia, dysmenorrhea, and non-menstrual pain [56]. Despite limited evidence of their efficacy and a lack of license for this specific indication [55], COCs have been widely used cyclically or continuously to treat endometriosis-associated symptoms; this is thought to be related, at least in part, to their non-endometriosis-specific benefits, including contraceptive protection and control of the menstrual cycle [57,58,59]. 

However, COCs may not be appropriate for all patients and are contraindicated in women older than 35 years who smoke or are at increased risk of myocardial infarction, stroke, or venous thromboembolism [55]. Furthermore, as endometriosis is highly estrogen dependent [60,61], supplementation of endogenous estrogen through the use of estrogen-containing COCs may cause exacerbation of the disease [62]. Additionally, while COCs are effective in thinning the eutopic endometrium, insufficient evidence is available of its effectiveness in diminishing the activity of endometrial implants [55,63,64,65]. In the context of these limitations, oral progestin-based therapies are generally a better option compared with COCs for the medical management of endometriosis, as oral progestins are not contraindicated according to patient age and smoking status; neither increase the risk of thrombosis nor induce amenorrhea; and have a generally favorable tolerability profile [55].

### 3.5. Consensus: Dienogest Can Be Used Long-Term If Needed

Endometriosis is typically considered a chronic disease [66,67], which therefore may require a lifelong management plan [21]. The use of medical treatment to avoid repeated surgical procedures is recommended, as surgeries are associated with inherent risks and repeated procedures might lead to pain-causing adhesions and adversely affect ovarian reserve [22]. Therefore, patients may require long-term medical therapy. 

Dienogest is a selective progestin that combines the pharmacological properties of 19-nortestosterone and derivatives of progesterone, with high specificity for progesterone receptors and minimal androgenic, estrogenic, glucocorticoid, and mineralocorticoid activity [23,68]. Additionally, dienogest has in vitro anti-inflammatory and progesterone receptor upregulation activity, supporting its efficacy in improving patient response to medical management [69,70,71]. 

Currently, long-term follow-up for dienogest is at least 60 months in clinical studies that include those from Japan and patients from adolescence to women in their fifth decade (Table 2) [72,73,74,75,76,77,78,79,80]. A 5-year study found that dienogest (2 mg/day) effectively reduced endometriosis-associated pelvic pain and avoided pain recurrence post-surgery [78]. The treatment was well tolerated with clinically manageable adverse effects. Dienogest was also reported to decrease recurrence after endometrioma excision, although metrorrhagia and decreased bone mineral density (BMD) were observed [79].

### 3.6. Consensus: A Large Evidence Base Exists Supporting the Use of Dienogest Compared with GnRH agonists as First-Line Medical Therapy for Endometriosis

A systematic review of 9 RCTs comparing dienogest to other medical therapies for endometriosis treatment found that dienogest was significantly better than placebo and as effective as GnRH agonists in reducing pelvic pain symptoms. Dienogest was also effective in reducing endometriotic lesions and frequency of hot flushes [68]. However, there was a higher frequency of irregular vaginal bleeding with dienogest compared with GnRH agonists. These results are generally consistent with another systematic review of 5 RCTs of dienogest versus placebo and GnRH agonists [81]. Notably, this second systematic review found that dienogest and buserelin intranasal spray appeared equally effective in improving QoL, but the comparative QoL effects of dienogest with other GnRH agonists could not be determined as no RCTs meeting the authors’ inclusion criteria considered this comparison. However, a RCT of 24 weeks of dienogest versus leuprolide acetate found a pronounced improvement in QoL measures with dienogest [82]. 

### 3.7. Consensus: GnRH Agonists May Be Considered for First-Line Therapy Only in Some Specific Situations or as Short-Term Therapy before Dienogest

GnRH agonists bind to receptors in the pituitary gland, thereby downregulating the pituitary–ovarian axis and causing hypoestrogenism [22], with the subsequent induction of amenorrhea and progressive endometrial atrophy thought to inactivate pelvic lesions and relieve endometriosis-associated pain [7,8,10,22,83]. However, GnRH agonists cause symptoms of estrogen deficiency, including BMD depletion as well as breakthrough bleeding, vaginal dryness, irritability, fatigue, headaches, depression, and skin problems [7,22]. Given the chronic nature of endometriosis, the adverse effects of GnRH agonists preclude its long-term use for this indication, and there is insufficient evidence of the benefits of using lower GnRH agonist doses (i.e., ‘draw-back’ therapy) [51,83]. Accordingly, we only recommend short courses of GnRH agonist therapy because of the risk of BMD loss. Additionally, we recommend hormonal add-back therapy to prevent bone loss and hypoestrogenic symptoms during GnRH agonist treatment. This recommendation is supported by data from a prospective, non-randomized trial of women with chronic pelvic pain associated with recurrent endometriosis, who achieved pelvic pain relief after 4–6 months of treatment with a GnRH agonist followed by 12 months of therapy with dienogest (1–2 mg/day) [84]. The use of GnRH agonists in young women and adolescents who have not reached maximum bone density requires careful consideration [7,10,26]. Therefore, we recommend that GnRH agonists be considered as a first-line, short-term therapy only for carefully selected patients.

### 3.8. Consensus: NSAIDs May Be Considered as Add-on Therapy for Endometriosis-Associated Pelvic Pain

Limited evidence exists regarding the use of NSAIDs for endometriosis treatment, apart from a single trial of NSAIDs versus placebo that found no evidence of a beneficial pain-relieving effect of NSAIDs in 24 women with endometriosis [85]. However, the favorable effect of NSAIDs on primary dysmenorrhea [86] supports its use for analgesia of endometriosis-associated pain, and may be considered particularly for young patients solely with dysmenorrhea and the absence of other endometriosis symptoms. 

Limitations of NSAIDs include potential inhibition of ovulation, the risk of gastric ulceration and cardiovascular disease, and their inability to alter the disease course [85,86,87]. Additionally, NSAIDs are generally insufficient for treatment of patients with a confirmed diagnosis of endometriosis or with symptoms other than dysmenorrhea. Therefore, we recommend that NSAIDs may be considered only as add-on, short-term therapy for endometriosis-associated pelvic pain.

## 4. Conclusions

This review and consensus deliberations considered clinical diagnosis and early medical management of endometriosis within Asia. The diagnosis and treatment of endometriosis are evolving, with a greater emphasis on patient-centered care that includes clinical diagnosis and early medical management [21]. Furthermore, new therapies for endometriosis are available and several others are in development. This changing paradigm for clinical diagnosis and medical management of endometriosis necessitates consideration of how best to deliver patient-centered care to women with endometriosis. 

**Figure 1:**
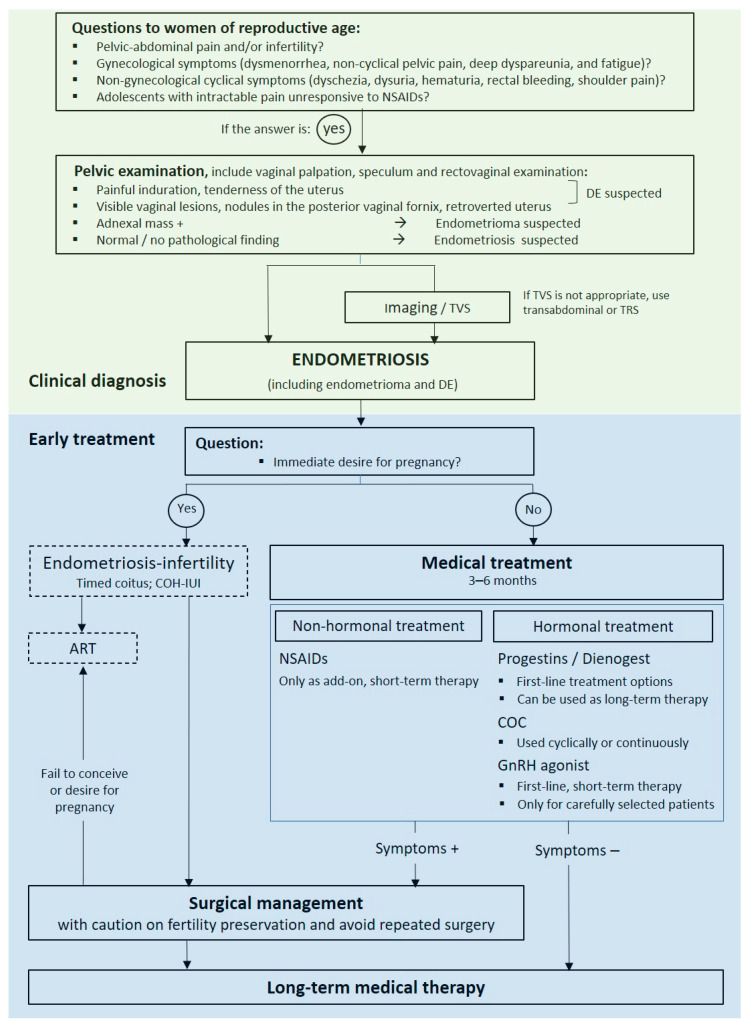
Algorithm for the clinical diagnosis and early treatment of endometriosis in Asia. ART, assisted reproductive technology; COC, combined oral contraceptive; COH, controlled ovarian hyperstimulation; DE, deep endometriosis; GnRH, gonadotropin-releasing hormone; IUI, intra-uterine insemination; NSAID, nonsteroidal anti-inflammatory drug; TRS, transrectal sonograph; TVS, transvaginal sonography.

The evolving paradigm emphasizes the importance of early clinical diagnosis. However, although clinical diagnosis is used in practice [14], the approach has not been standardized [11]. A consistent approach to clinical diagnosis and treatment is necessary to optimize patient care and outcomes. A validated algorithm that utilizes both clinical diagnosis and early medical therapy using contemporary treatment approaches is not available currently. Based on our consideration of the available evidence from recent international guidelines and consensus reports and the literature on clinical diagnosis and early medical management of endometriosis, we propose an algorithm that incorporates clinical diagnosis and early medical management for endometriosis in Asia (Figure 1). Notably, further evaluation of such an algorithm and incorporation into routine practice will require consideration of its effect on diagnosis rates and patient outcomes. Additionally, because the role of surgery and medical management before surgery and the role of medical management before assisted reproductive technology (ART) are important aspects of the patient journey (i.e., the ‘endometriosis life’ [21]), these were included within the algorithm, but their in-depth consideration were beyond the scope of our review, which focused on clinical diagnosis and early medical management of endometriosis. 

The strength of our work is that it provides a contemporary assessment of current practice and treatments. Additionally, our recommendations are based on the results of a consensus meeting of many specialists across several Asian countries, which was the first consensus meeting that we are aware of spanning several Asian countries and addressing clinical diagnosis and early medical therapy; we note from our clinical experience that this concept has been recently accepted in Asia. 

Limitations of the available dataset for clinical diagnosis and early medical management are noted. For instance, the duration of follow-up of many studies is limited and few comparative studies of medical management, or combined medical treatments, and of studies within Asian populations are available. Additionally, as described above, important aspects of the journey for a patient with endometriosis were not considered and warrant future consideration within the Asian population, including the role of surgery, the importance of medical management before ART, and medical treatment of specific phenotypes (e.g., extra-genital endometriosis). 

In conclusion, in the context of the changing paradigm of diagnosis and management, this consensus guidance recommends that early clinical diagnosis and medical treatment of endometriosis be considered, including within Asia, as a means of delivering patient-centered care to women with endometriosis.

## Figures and Tables

**Table 1:** Guidelines and consensus reports referred to in the development of the current consensus recommendations for Asia.

Group	Title	Reference
**Global**		
World Endometriosis Society Montpellier Consortium	Consensus on current management of endometriosis	Johnson et al., 2013 [8]
**European**		
The European Society of Human Reproduction and Embryology (ESHRE)	ESHRE guideline: management of women with endometriosis	Dunselman et al., 2014 [10]
National Institute for Health and Care Excellence (NICE)	Endometriosis: diagnosis and management	NICE 2017 [24]
National German Guideline	National German guideline (S2k): Guideline for the diagnosis and treatment of endometriosis	Ulrich et al., 2014 [25]
**North American**		
American Society for Reproductive Medicine (ASRM)	Treatment of pelvic pain associated with endometriosis: a committee opinion	The Practice Committee of the American Society for Reproductive Medicine 2014 [22]
**Asian**		
Korean Society of Endometriosis (KSE)	Clinical evaluation and management of endometriosis: guideline for Korean patients from Korean Society of Endometriosis	Hwang et al., 2018 [26]
Obstetrical and Gynaecological Society of Malaysia	Clinical guidelines for the management of endometriosis 2016	Subramaniam et al., 2016 [7]
The Federation of Obstetric and Gynaecological Societies of India (FOGSI)	Good Clinical Practice Recommendations on Endometriosis	FOGSI 2017 [27]

**Table 2:** Overview of studies supporting long-term treatment with dienogest.

Study	Population (Age)	Intervention ^a^ (Setting)	Treatment Length	Outcomes
Cosson et al. [77]	*n* = 130 (mean: 28.5–30.3 y)	DNG vs. GnRH ^b^ (post-surgical consolidation therapy)	16 wks	▪ VAS not significantly different between groups ▪ AEs reported by 87.7% of DNG- and 85.1% of GnRH-treated patients
Harada et al. [76]	*n* = 271 (mean: 33.5–33.8 y)	DNG vs. GnRH ^c^	24 wks	▪ DNG reduced all subjective symptom scores; all mean changes with DNG were comparable to those obtained with GnRH apart from induration in the POD ▪ DNG associated with less hot flushes and BMD loss than GnRH, but associated with more irregular genital bleeding
Strowitzki et al. [75]	*n* = 229 (mean: 30.6–31.0 y)	DNG vs. GnRH ^d^	24 wks	▪ Treatment difference in VAS favored DNG; non-inferiority of DNG relative to GnRH was shown (*p* < 0.0001) ▪ DNG associated with less hypoestrogenic effects than GnRH, but with more bleeding episodes ▪ DNG had less BMD effects than GnRH (0.25% vs. −4.04%; *p* = 0.0003)
Köhler et al. [74]	*n* = 68 (mean: 27.6–33.5 y)	DNG ^e^ (dose-finding study)	24 wks	▪ Mean revised AFS scores reduced in the 2 mg/day and 4 mg/day groups ▪ DNG generally well tolerated with low rates of AE-related discontinuations
Momoeda et al. [73]	*n* = 135 (mean: 34.1 y)	DNG	52 wks	▪ 72.5% and 90.6% of patients had global improvement of subjective symptoms at 24 and 52 wks, respectively ▪ No clinically significant changes seen in incidence or severity of AEs over treatment course ▪ Statistically significant decrease in BMD seen at 24 and 52 wks (−1.6% and −1.7%, respectively), but no cumulative decreases observed
Petraglia et al. [72]	*n* = 152 (18–45 y ^f^)	DNG	36–52 wks	▪ Significant decrease in pelvic pain (*p* < 0.001) ▪ Mean frequency and intensity of bleeding decreased ▪ 16.1% of patients experienced potentially drug-related AEs; 92.5% were mild-to-moderate in severity
Ebert et al. [80]	*n* = 111 (adolescents; median [range] 16.0 [12,13,14,15,16,17] y)	DNG	52 wks	▪ Mean endometriosis-associated pain score decreased from 64.3 at baseline to 9.0 at 48 wks ▪ Mean relative BMD change from baseline to the end of study was −1.2%
Römer [78]	*n* = 37 (39 y)	DNG	60 mo	▪ EAPP was reduced and post-surgical pain recurrence was avoided ▪ 7 cases of spotting and 4 cases of depressed mood, which were clinically managed
Ota et al. [79]	*n* = 151 (32.6 y)	DNG vs. no therapy (post-surgical therapy)	60 mo	▪ Post-surgical recurrence rates were 4% with DNG and 69% with no therapy ▪ BMD decrease and depression were seen in 4% and 2.6% of patients with DNG, respectively

AE, adverse event; AFS, American Fertility Society; BMD, bone mineral density; DNG, dienogest; EAPP, endometriosis-associated pelvic pain; GnRH, gonadotropin-releasing hormone; POD, pouch of Douglas; VAS, visual analog score. ^a^ DNG was administered at a dose of 2 mg/day unless otherwise indicated. ^b^ Triptorelin 3.75 mg/month. ^c^ Intranasal buserelin acetate 900 µg/day. ^d^ Leuprolide acetate 3.75 mg/month. ^e^ 1, 2, or 4 mg/day. ^f^ Inclusion criteria.

## Data Availability

Not applicable.
